# Natural or Artificial? Habitat-Use by the Bull Shark, *Carcharhinus leucas*

**DOI:** 10.1371/journal.pone.0049796

**Published:** 2012-11-16

**Authors:** Jonathan M. Werry, Shing Y. Lee, Charles J. Lemckert, Nicholas M. Otway

**Affiliations:** 1 Australian Rivers Institute and School of Environment, Griffith University, Gold Coast, Queensland, Australia; 2 Ocean and Coast Research, Gold Coast, Queensland, Australia; 3 Griffith School of Engineering, Griffith University, Gold Coast, Queensland, Australia; 4 New South Wales Department of Primary Industries, Port Stephens Fisheries Institute, Taylors Beach, New South Wales, Australia; The Australian National University, Australia

## Abstract

**Background:**

Despite accelerated global population declines due to targeted and illegal fishing pressure for many top-level shark species, the impacts of coastal habitat modification have been largely overlooked. We present the first direct comparison of the use of natural versus artificial habitats for the bull shark, *Carcharhinus leucas*, an IUCN ‘Near-threatened’ species - one of the few truly euryhaline sharks that utilises natural rivers and estuaries as nursery grounds before migrating offshore as adults. Understanding the value of alternate artificial coastal habitats to the lifecycle of the bull shark is crucial for determining the impact of coastal development on this threatened but potentially dangerous species.

**Methodology/Findings:**

We used longline surveys and long-term passive acoustic tracking of neonate and juvenile bull sharks to determine the ontogenetic value of natural and artificial habitats to bull sharks associated with the Nerang River and adjoining canals on the Gold Coast, Australia. Long-term movements of tagged sharks suggested a preference for the natural river over artificial habitat (canals). Neonates and juveniles spent the majority of their time in the upper tidal reaches of the Nerang River and undertook excursions into adjoining canals. Larger bull sharks ranged further and frequented the canals closer to the river mouth.

**Conclusions/Significance:**

Our work suggests with increased destruction of natural habitats, artificial coastal habitat may become increasingly important to large juvenile bull sharks with associated risk of attack on humans. In this system, neonate and juvenile bull sharks utilised the natural and artificial habitats, but the latter was not the preferred habitat of neonates. The upper reaches of tidal rivers, often under significant modification pressure, serve as nursery sites for neonates. Analogous studies are needed in similar systems elsewhere to assess the spatial and temporal generality of this research.

## Introduction

Identifying spatial and temporal patterns of abundance, reproduction, demography and capacity to withstand exploitation through directed fisheries or destruction of essential habitat is critical for managing the conservation of sharks. Dramatic global declines in shark populations in oceans and near-shore areas [1], [2], [3], have been attributed to recognised fisheries, illegal unregulated fishing [4], [5], and the demand for shark-fins fuelled by booming Asian economies [6]. For many sharks, these pressures are exacerbated by life history characteristics, comprising slow growth, late onset of sexual maturity and low fecundity [7], [8], [9]. However, the impacts of habitat destruction/modification on coastal shark species are relatively untested despite the recognised value of estuaries as essential space-limited nursery habitats for many neonate and juvenile sharks [10], [11], [12]. With the recent significant coastal urbanisation, habitat destruction is accelerating [13], [14]. The cumulative effects of this impact may have far more wide-reaching ramifications for shark populations, particularly if juveniles are removed from coastal areas before they are able to mature and reproduce.

Large-scale urban developments, particularly canals and residential canal estates, occur throughout the world and these simultaneously destroy natural habitats and create artificial estuarine habitats that may mimic the biologically diverse and productive natural ecosystems [15], [16]. The natural estuarine habitats provide nurseries for many commercially important species, including prawns, crabs, fish and sharks [17], [18], [19], and are ecologically important to various stages in the lifecycle of sharks (e.g. [20], [21], [11]). Surprisingly, artificial estuarine habitats often have similar fish communities and provide prey for predators including sharks and rays [22], [23], [24]. They also enable inter-changeable habitat-use in response to ontogenetic changes and environmentally driven movements.

Monitoring such movements in coastal environments has been greatly advanced through passive acoustic telemetry [25], [26], [27], [28], which has been successfully applied to determining the occurrence and movements of sharks in estuaries [29], [30], [31]. More recent studies have used this powerful tool to quantify the ontogenetic changes in movement, home range and habitat-use of blacktip [32], bonnethead [33], lemon [34], [21], leopard [35], pig-eye [11], sandbar [36], and bull sharks [21], [12].

The bull shark (*Carcharhinus leucas*) is a cosmopolitan species that grows to almost 4 metres [37], [38], and exhibits a global distribution mirroring that of residential canal estates [16]. Bull sharks utilise a wide range of salinities throughout their lifecycle starting with neonates in low salinity nursery habitats [39], [40], juveniles in riverine/estuarine habitats [20], [12], and adults in coastal, marine waters off south Africa [41], Florida [42], [43], [44], Fiji [45], and Australia [46], [12]. Despite its wide distribution, the bull shark is now considered ‘Near-threatened’ globally on the IUCN Red List as a direct result of anthropogenic impacts such as habitat modification and targeted/indiscriminate fishing [47], [48].

**Figure 1:**
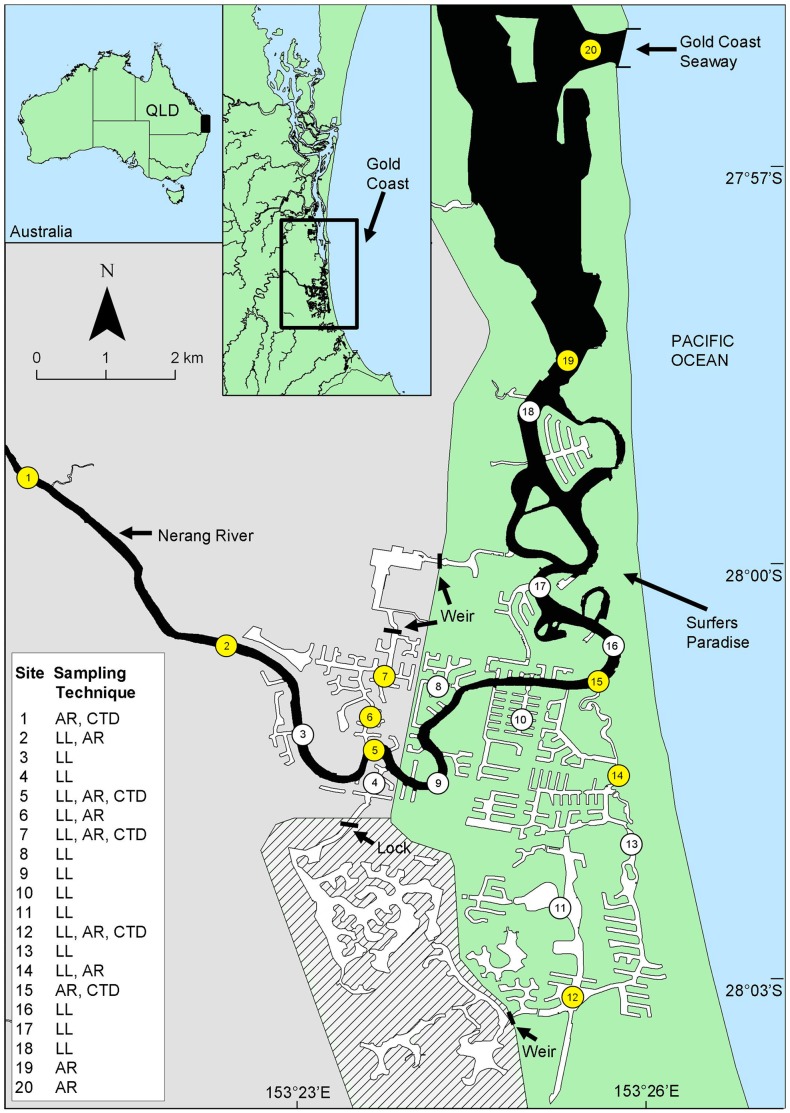
Gold Coast Canal system. Map illustrating the location of the Nerang River (natural habitat) and Broadwater (in black) and adjoining canal systems (artificial habitat) (in white). Green and grey section of main map illustrates the approximate delineation of low (grey) and high (green) saline areas and upper (grey) and lower (green) river and canals. Numbers indicate the locations of longline surveys (LL), acoustic receivers (AR- highlighted in yellow) and conductivity temperature and depth meters (CTD). Lined area indicates freshwater separated from the artificial and natural habitats by a weir and lock.

In Australia, bull sharks mainly occur in Australia’s tropical and sub-tropical coastal waters, estuaries and rivers [49], [50], [51]. In SE Queensland (QLD), juvenile bull sharks occur in freshwater/estuarine regions [52], [12], whereas the adults are found in nearshore, coastal habitats [53], [54], [12]. This pattern arises via the differential use of various natural habitats by particular ontogenetic stages in the lifecycle of bull sharks [12]. Furthermore, given the species’ osmoregulatory capabilities, it is not surprising that bull sharks have occupied man-made habitats including an impoundment in Panama [55]. Unfortunately, little is known about the species’ use of man-made waterways in urbanised coastal regions throughout the world [37], [56], and this is also true for SE QLD, where substantial urbanisation has occurred with residential canal estates linked to natural waterways. A clear impetus for our research stemmed from past and present events in the Gold Coast region (Fig. 1) including: two fatal shark attacks on swimmers in December 2002 and February 2003, numerous recent shark sightings and continuing media reports [53]. Understanding the degree to which bull sharks use the man-made habitats and if this differs with the various ontogenetic stages will be necessary for mitigating the risks of attack in the future and will be important for managing the long-term conservation of bull sharks in Australia’s progressively urbanised coastal environments. Hence, this study documents the movements, occupancy patterns and associated salinity ranges of bull sharks in a natural river habitat, and the adjoining, man-made canals and in so doing, for the first time, contribute to testing the general null hypothesis of no difference in the usage of natural and artificial habitats by neonate and juvenile bull sharks.

## Materials and Methods

### Ethics Statement

This research was done in accordance with QLD Fisheries permit 90306 and a Griffith University animal care and ethics approval EAS/05/05/AEC.

### Study Sites

Our study focussed on the Gold Coast, Australia’s fastest growing city, where increased urbanisation is centred on man-made canals linked to the natural Nerang River (Fig. 1) and produced the Gold Coast System (GCS). The Nerang River is subjected to periodic flooding, non-linear tidal forces and a semi-diurnal tide [57]. Its mouth at the Broadwater (Fig. 1) is ∼ 250 m wide, 3–5 m deep, has a mean tidal range of 1.2 m and salinity ranging from 19 to 32 (PSU). Along the river’s path are several deep (5–10 m) holes, and intermittent stands of mangroves, mainly *Avicennia marina* and to a lesser extent *Aegiceras corniculatum* and *Rhizophora stylosa*. The upper estuarine region is ∼ 11 km from the mouth, has salinities ranging from 6 to 18, a mean tidal range of 0.72 m and depths dropping to ∼ 1.0 m below low water. The river is connected at several locations to the 200 linear km network of tidal canals that vary in width and depth (15–100 m and 0.5–15 m, respectively) [58], and are used throughout the year for recreational activities including boating, water-skiing, fishing and swimming. While the canals are inhabited by an estuarine fish community with trophodynamics that differ from natural habitats [59], [60], the species composition overlaps [61], and provides adequate prey for bull sharks [62], [12].

The combined area of low salinity (6–18) waters was estimated as 165.17 km^2^ (comprising 82.60 and 82.57 km^2^ for natural and artificial habitats, respectively) and compared to 569.07 km^2^ (comprising 220.32 and 348.75 km^2^ for natural and artificial habitats, respectively) for that of the higher salinity environment (19–32). The natural habitat provided 302.92 km^2^ of available area for the different ontogenetic stages of the bull shark as compared to 431.32 km^2^ for the artificial (canal) habitat.

### Patterns of Relative Abundance

To quantify the spatial and temporal patterns of relative abundance and size-composition of different ontogenetic stages of the bull shark in the Nerang River and adjoining canals, modified longlines were set at 16 sites (7 in the Nerang River, 9 in the canals) on a quarterly basis over 3 years from 2006 to 2008 (Fig. 1). Each longline comprised two 8/o, offset tuna hooks (Mustad, Gjovik, Norway) baited with freshwater eel and mullet attached to 1 m long stainless wire traces with one suspended from a surface float and the other approximately one metre above the seabed. Each site was sampled over three consecutive nights per quarter with the lines set between 17∶00 and 19∶00 in a random sequence on each occasion across the 16 sites and allowed to fish for approximately two hours prior to checking. Most sharks caught were tagged with individually-numbered spaghetti tags (Hallmark™) to permit identification of any recaptured animals, whereas some individuals were tagged with acoustic tags (see below).

### Acoustic Tagging

Bull sharks were captured using the modified longlines at the 16 sites distributed across the natural and man-made habitats (see above). Fishing with a rod and line was used to supplement the catches of bull sharks in the upper Nerang River. Moreover, a previous study [63] has shown that bull sharks exhibit less capture-stress compared to other species when caught using gillnets, we also deployed an 8 cm stretch-mesh gillnet for 30 minutes on various occasions in the uppermost reaches of the Nerang River to provide additional animals for tagging. All sharks caught were restrained in a harness alongside an anchored 4.5 m research vessel that was orientated to maximise the tidal flow of water over the shark’s gills [12]. Duration of restraint, especially if prolonged, can affect the blood pressure and acid-base balance of sharks and increase their subsequent recovery times [64], [63], [65], [66]. Hence, tagging, length measurements and hook removal were completed within 20 minutes while the sharks were restrained in the harness, in dorsal recumbency to induce tonic immobility [67] and reduce struggling and stress (see below). Each shark was tagged with either a Vemco V16 or V13 R-coded, 69 kHz acoustic tag (Amirix Systems Inc., Nova Scotia, Canada) that transmit a unique identification number, has battery lives of 24 and 34 months, and acoustic ranges of 800 m and 400 m, respectively, given average coastal, sea-conditions and wind-strengths of 11–16 knots (20–29 km/hr) (www.vemco.com/education/range.php). Each acoustic tag was glued into a float to prevent chaffing of the underlying skin [68] and then fixed to the pin of a numbered jumbo rototag (Dalton Ltd, UK) using 100 kg breaking-strain monofilament. The cattle ear tag was then attached to the first dorsal fin using a hole-punch and a standard tag applicator. While sharks have a natural bacterial flora on their skin [69], alcohol was not used to sanitise the skin around the tagging site because it can result in localised erythema, induration, and excess mucus [70], [71], [72]. We deliberately chose external tag attachment, in contrast to surgical implantation (e.g. [31], [12]), to enhance re-sighting and reporting by local residents and recreational fishers, some of whom target bull sharks throughout this system [73]. Precaudal and fork lengths were then measured to the nearest cm and converted to total length (TL) using significant linear regression relationships. Finally, the hook was removed and the shark released.

Capture stress is an integrated response and often greater in neonates and juveniles [74]. Sharks exhibiting signs of capture stress and/or restraint often become much lighter in colour due to vasoconstriction of peripheral blood vessels and this provides a reliable, visual indicator [75], [76]. Thus, we recorded skin colour and, following release, the swimming behaviour of each shark.

### Movements and Habitat-use

The movements of the acoustically-tagged sharks and their duration of occupation of natural and artificial habitats were quantified over 16 months from February 2007 to May 2008 using an array of 10 Vemco VR2/VR2W omni-directional acoustic receivers (Amirix Systems Inc., Nova Scotia, Canada) strategically deployed as gates ensuring detection (*sensu* Heupel *et al.* [28]) at various sites (Fig. 1). Five receivers were deployed in the Nerang River, two in each of the upper and lower reaches, and one centrally-located in the mouth of the river. Four receivers were deployed in the canals, two in each of the upper and lower regions of differing salinities (Fig. 1). The maximum tag ranges, habitat complexity, and acoustic receiver sites (Fig. 1) meant that the presence of each tagged bull shark was unequivocally documented. Prior to deployment, each acoustic receiver was wrapped with duct tape and then coated with a copper-based antifouling paint to prevent the growth of fouling organisms that can reduce acoustic detection efficiencies [77]. Each receiver was attached to a navigation marker or jetty piling approximately 1 to 3 m below mean low water mark. Retrieval, data download and replacement of the acoustic receivers was done at ∼ 2-monthly intervals. Following data download, the detections were sorted by shark ID, ontogenetic stage, site, date and time. This permitted the documentation of the timing and duration of occupation of particular locations, diurnal patterns and the movements of individuals among sites. As detailed analyses of active and passive acoustic telemetry of bull sharks will be described elsewhere, only representative examples of the movements of individuals among acoustic listening station sites are provided here. Moreover, as this study focused on the usage of this system at larger spatial scales, the acoustic detection data were pooled across replicate acoustic telemetry sites within the natural and artificial habitats with low and high salinities. Finally, salinity and water temperatures were also recorded whenever possible at five sites (Fig. 1) using CTD profilers (Greenspan CTD350) to provide contemporaneous data augmenting the previous, detailed information collected for hydraulic-modelling and flood-mitigation studies [78], [79], [80].

### Statistical Analyses

All analyses were done using SPSS v17 (Armonk, NY, USA), Genstat 13 (Hemmel Hempstead, UK) and Datadesk v6 (Data Description Inc.) software. Heteroscedasticity was examined using Cochran’s test and when necessary data were transformed in accordance with the recommendations of Snedecor and Cochran and Underwood. Significant differences among means were identified using Student-Newman-Keuls (SNK) tests. The proportions of neonate (0.50 to 0.84 m TL) and juvenile (0.85 to 1.6 m TL) bull sharks caught in the upper and lower Nerang River, and the adjoining upper and lower canals pooled over three years (i.e.12 quarterly sampling periods) were analysed using a χ^2^ test. Differences in catch per unit effort (CPUE, expressed as the number of sharks caught per 50 hooks) of bull sharks pooled over the three years were examined using a fully-fixed, 2-factor analysis of variance (ANOVA) with the factors Seasons and Habitats each with 4 levels. Habitats comprised the Nerang River (Natural) and the Canals (artificial) and each had 2 levels comprising the upper and lower areas, respectively. The mean TL of bull sharks caught in the quarterly surveys in the upper and lower areas of each habitat were analysed using unbalanced, 1-factor analyses of variance (ANOVA). The mean TL of bull sharks captured on the surface or bottom baited hooks was compared using a t-test.

Comparisons of the proportions of neonates and juveniles caught and tagged in the Nerang River and adjoining canals, together with possible differences from 1∶1 sex ratios were analysed using χ^2^ tests. The greatest distance moved by each tagged shark detected on the various receivers was plotted against TL to examine whether the maximal displacement of each tagged shark was related to its size with Pearson’s correlation coefficient (*r*) calculated to test the significance of relationship. The acoustic detections were also used to determine the proportion of time that neonate and juvenile bull sharks spent in areas of low or high salinity and the proportion of time spent in natural (Nerang River) or man-made (canals) habitats. To account for differing transmission rates acoustic detections were placed into 15 minute bins. These data were then analysed using paired and unpaired *t*-tests following arcsine transformation.

The acoustic detections were also used to determine if individual neonate and juvenile bull sharks selected or avoided high or low salinity waters and/or natural (river) and man-made (canal) habitats. The preference for low salinity areas and natural habitats were compared to that available using Chesson’s α [81] where α ranges from 0 to 1 with values close to 1 indicating electivity. The data were processed in a similar manner to Heupel and Simpfendorfer [10] with salinity data recorded at five sites (Fig. 1) and modelled (via Generalised Linear Modelling) for 15 minute blocks for the entire system over the period of acoustic monitoring. The areas (km^2^) of natural and man-made habitats were calculated using GIS (ArcView version 9.3). The cumulative time spent by individuals in natural and/or man-made habitats were determined from the binned acoustic detections and compared with the respective areas of available habitat determined using GIS. Electivity for low saline areas and natural habitat were calculated and plotted for neonate and juvenile bull sharks of varying TL.

## Results

### Relative Abundance Patterns

Sixty-six bull sharks were caught over the three years of sampling using longlines, gillnets, and rod and line (Table 1). The vast majority of these (n = 49) were caught on longlines with 14 (6 neonates, 8 juveniles) caught in the upper reaches of the Nerang River and a further 13 individuals (11 juveniles, 1 sub-adult male, 1 pregnant female) caught in the lower reaches. The remaining 22 bull sharks (3 neonates, 19 juveniles) were caught in the canals with the neonates only caught in the upper canals. Juveniles were caught in the upper (n = 6) and lower (n = 13) canals, respectively. A further 9 individuals (8 neonates, 1 juvenile) were caught in gillnets in the upper reaches of the Nerang River. The remaining 8 juvenile bull sharks were caught in the upper and lower reaches of the Nerang River and in the lower canals on a rod and line. The proportion of neonate and juvenile bull sharks caught on longlines differed significantly within habitats (χ^2^ = 11.94, df = 3, p = 0.0076) with proportionally more neonates caught in the upper reaches of the Nerang River and associated canals, and proportionally more juveniles caught in the lower reaches of the Nerang River and its adjoining canals. A similar result was also evident for neonates and juveniles caught using all three sampling techniques (Table 1) and (χ^2^ = 12.72, df = 3, p = 0.00025). However, when catches were pooled over reaches/areas (i.e. upper and lower) within habitats, the proportions of neonate and juvenile bull sharks caught on longlines did not differ significantly between habitats (χ^2^ = 0.37, df = 1, p = 0.81) and this was mirrored with all sampling techniques (Table 1) and (χ^2^ = 3.36, df = 1, p = 0.067).

**Table 1:** Total length and ontogenetic stage of bull sharks in the Gold Coast System.

Habitat		Seasons			
		Spring	Summer	Autumn	Winter
RU	Mean TL(± SE)	109.66 (6.30)	85.19 (2.91)	85.24 (6.73)	94.00 *
	Range (cm)	96–122	75–109	49–113	–
	Stage	J	N, J	N, J	J
CU	Mean TL(± SE)	–	82.77 (1.54)	85.00 (1.55)	–
	Range (cm)	–	79–85	79–87	–
	Stage	–	N, J	N, J	–
RL	Mean TL(± SE)	111.49 (6.55)	89.93 (5.09)	126.90 (12.16)	–
	Range (cm)	89–127	85–95	93–181	–
	Stage	J, A^#^	J	J, SA	–
CL	Mean TL(± SE)	114.87 (14.68)	117.23 (17.16)	114.30 (6.12)	–
	Range (cm)	86–133	89–148	92–142	–
	Stage	J	J	J	–

Mean (±SE) total length (TL) and range of *C. leucas* of different ontogenetic stages (N = neonate, J = juvenile, SA = subadult, A = adult) caught on rod and line (n = 8), longlines (n = 49) and in gillnets (n = 9) in the upper and lower areas of the Nerang River (natural habitat) and the upper and lower canals (man-made habitat) from January 2006 to December 2008. (RU = river upper, CU = canal upper, RL = river lower, CL = canal lower). Note: rod and line fishing done sporadically across the entire system and gillnets were only deployed in the upper reaches of the Nerang River. Note: A^#^ = a putatively pregnant female 300 cm TL.

**Figure 2:**
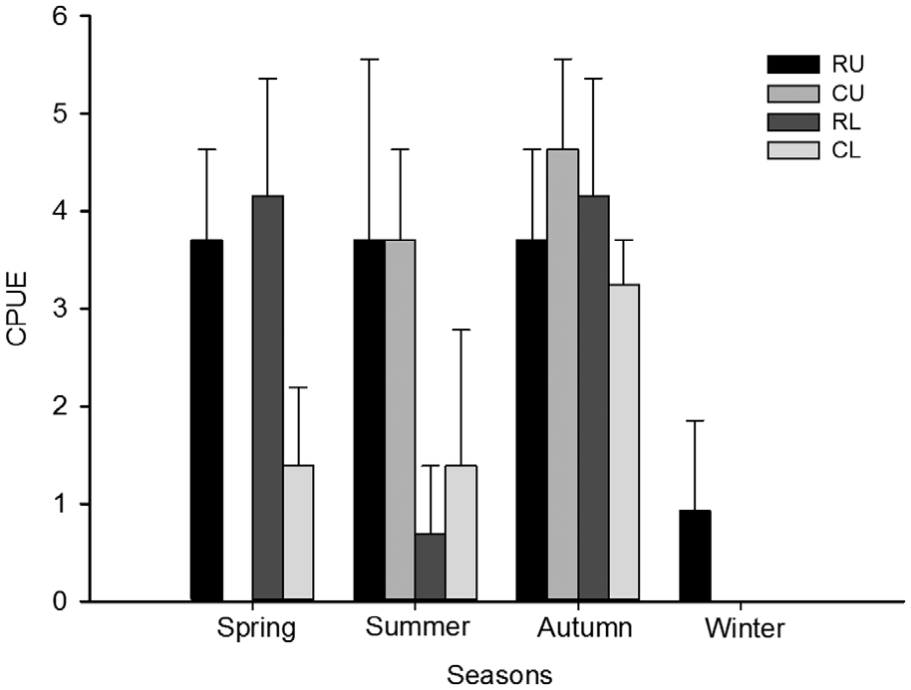
Catch per unit effort (CPUE) (± SE) for quarterly longlines surveys for bull sharks in the Gold Coast System. Quarterly surveys for 2006–2008 in natural and artificial habitats. RU = river upper, CU = canal upper, RL = river lower, CL = canal lower.

The CPUE of bull sharks on longlines (Fig. 2) differed significantly among Seasons (F_3, 32_ = 10.65, p = 0.001), but not among Habitats (F_3,32_ = 1.78, p = 0.17) and was maximal in autumn, which was significantly greater than that in spring and summer, which did not differ, but was significantly greater than that in winter (Fig. 2; SNK test: p = 0.05). Only one bull shark, a juvenile male (94 cm TL), was caught during the winter months over the three years of sampling. In spring, CPUE was greatest in the upper and lower reaches of the Nerang River reflecting the presence of mainly juveniles. While not statistically different from spring, the CPUE in summer was greatest in the upper reaches of the Nerang River and its adjoining canals reflecting the presence of juveniles and recently recruited neonates. CPUE in the lower river and adjoining canals, whilst not significant in summer, was lower and reflected the presence of juveniles. CPUE was similar across the natural and man-made habitats during autumn and reflected greater a greater dispersal of neonates and juveniles throughout the system.

The length-frequency distributions of bull sharks caught using longlines, gillnets, and rod and line in the upper and lower reaches of the Nerang River and its adjoining canals suggested that there were no differences in TL between sampling methods, but there appeared to be differences between ontogenetic stages and habitats: a result confirmed by analysis after excluding the sub-adult male and adult female bull sharks. The vast majority of bull sharks were caught on the bottom-set hooks (n = 37) compared to surface-set hooks (n = 12) and the mean TL did not differ significantly between the sharks caught at the bottom (105.7±3.5 cm TL) and at the surface (91.64±5.0 cm TL) over the three years of sampling (t-test: t = 1.96, df = 45, p>0.05). The mean TL of neonates caught in the gillnets did not differ significantly from those caught on longlines in the upper canals and upper reaches of the Nerang River (ANOVA: F_2,14_ = 2.13, p = 0.16). In contrast, the mean TL of juvenile bull sharks in the upper and lower reaches of the Nerang River (101.9±3.1 cm and 111.2±5.4 cm, respectively) and the lower canals (115.1±5.3 cm) were significantly greater than the mean TL of juveniles and neonates in the upper canals (86.1±0. 45 and 79.9±0.8 cm, respectively) and the neonates in the upper Nerang River (77.3±2.3 cm) (ANOVA: F_5,58_ = 12.86, p<0.001; SNK test: p<0.05). After pooling across ontogenetic stages, the overall mean TL of bull sharks caught still differed significantly among habitats (ANOVA: F_3,60_ = 11.99, p<0.001) with larger individuals occurring in lower reaches of the Nerang River and lower canals compared to the upper reaches of the river and its adjoining canals (SNK tests: p<0.05). Furthermore, a putatively pregnant female was caught midway between sites 9 and 15 (Fig. 1) in the lower reaches of the Nerang River in November 2008.

### Tagging and Capture Stress

**Figure 3:**
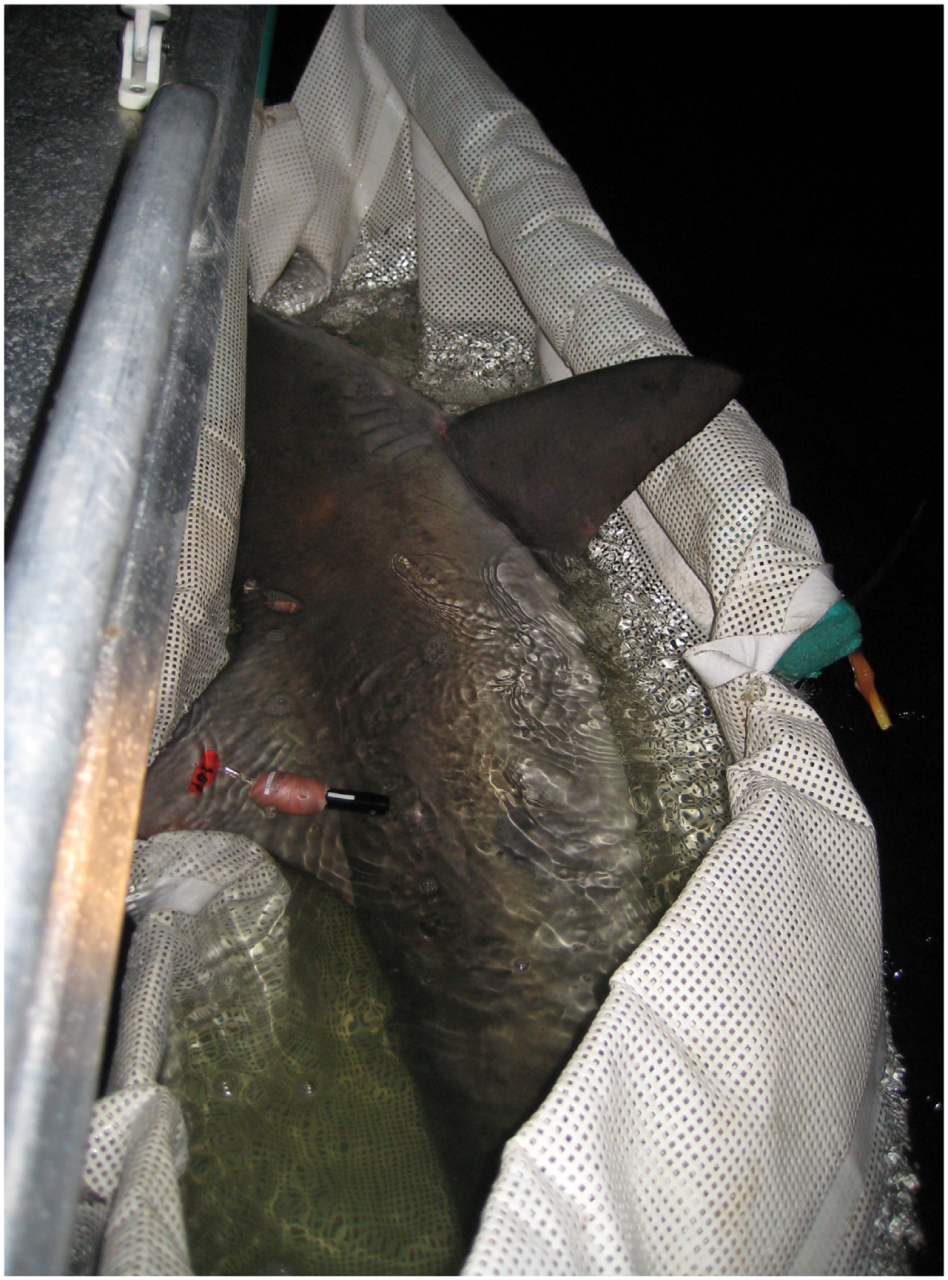
External acoustic tag and restraint of bull shark in harness.

None of the neonate or juvenile bull sharks were adversely affected by the stress of capture and their subsequent restraint (Fig. 3). This was evident by the eyes of each shark appearing normal with the left and right pupils equally reactive to bright light. The skin of each bull sharks was a natural grey colour and elastic at capture, and did not exhibit pallor or acquire a blotchy appearance. Moreover, the trunk muscles did not display any signs of rigidity during the period of restraint. Following release, all of the sharks swam away with vigor and displayed normal movements.

Twenty-four bull sharks comprising 10 neonates (mean TL ± SE: 6 male, 78.7±1.5 cm and 4 female, 79.8±0.9 cm) and 14 juveniles (mean TL ± SE: 6 male, 111.0±8.2 cm and 8 female, 108.4±8.4 cm) were tagged with acoustic tags between February 2007 and March 2008 (Table 2). The proportions of male and female sharks tagged did not differ significantly in 2007 and 2008 (χ^2^ = 3.00, df = 1, p = 0.08) nor between ontogenetic stages (χ^2^ = 0.69, df = 1, p = 0.41). Furthermore, the proportions of neonate and juvenile sharks tagged in 2007 and 2008 were not significantly different (χ^2^ = 0.09, df = 1, p = 0.77). In contrast, the proportions of neonate and juvenile sharks tagged in the Nerang River and the canals differed significantly (χ^2^ = 7.06, df = 1, p = 0.008) with proportionally more neonates tagged in the river and proportionally more juveniles tagged in the canals. The sex ratios of the bull sharks caught and tagged did not differ significantly from unity for neonates and juveniles (χ^2^ = 0.69, df = 1, p = 0.41) nor between years (2007 and 2008: χ^2^ = 3.00, df = 1, p = 0.083).

**Table 2:** Summary of overall movements of bull sharks tagged with acoustic tags in the Gold Coast System.

Shark ID	Yeartagged	Ontogeneticstage	TaggingHabitat	Sex	Total length(cm)	Summary of movements withinand/or between habitats
1	2007	N	R_U_	M	75	R_U_ → C_U_ → R_U_ → R_L_ → C_L_
2	2008	N	R_U_	M	75	R_U_ #
3	2007	N	R_U_	M	77	R_U_ → R_L_
4	2008	N	R_U_	F	78	R_U_ → R_L_ → R_U_ →C_U_ → R_U_ →R_L_ → R_U_ →
						C_U_ → R_U_ →R_L_ → C_L_ → R_L_→ R_U_ → C_U_
5	2007	N	R_U_	F	79	R_U_
6	2007	N	R_U_	M	79	R_U_ → R_L_ → C_L_ → R_U_ →C_U_
7	2007	N	R_U_	F	80	R_U_
8	2007	N	R_U_	M	82	R_U_ → C_U_
9	2008	N	R_U_	F	82	R_U_ → C_U_ → R_U_ → R_L_
10	2007	N	R_U_	M	84	R_U_
11	2007	J	C_U_	M	85	C_U_ → R_U_ → C_U_ → R_U_ → R_L_ → C_L_
12	2007	J	C_U_	F	87	C_U_ #
13	2007	J	C_U_	F	87	C_U_ → R_U_ → C_U_
14	2008	J	C_U_	F	87	C_U_ → R_U_ → C_U_ → R_U_ → R_L_
15	2007	J	R_U_	M	91	R_U_ → C_U_ → R_U_ → C_L_
16	2008	J	R_U_	F	97	R_U_ #
17	2008	J	C_L_	F	113	C_L_ → R_L_ → R_U_ → R_L_ → C_L_ → R_L_
18	2007	J	C_L_	M	115	C_L_ → R_L_ → R_U_ → C_U_ → R_U_ → R_L_ → C_L_
19	2007	J	R_L_	F	116	R_L_ #
20	2007	J	R_L_	M	117	R_L_ → R_U_ → C_U_ → R_U_ → R_L_ → C_L_
21	2007	J	C_L_	M	118	C_L_ #
22	2007	J	R_L_	F	127	R_L_ → R_U_
23	2008	J	R_L_	M	140	R_L_ → C_L_ → R_L_
24	2008	J	R_L_	F	153	R_L_ → R_U_ → C_U_ → R_U_ → R_L_ → C_L_

Movements within or between habitats of river (R) and canal (C) by different ontogenetic stages. N = neonate, J = juvenile. Tagging Habitat, RU = river upper, CU = canal upper, RL = river lower, CL = canal lower, M = male, F = female. # refers to bull sharks captured by recreational fishers.

### Movements Among Sites and Habitats

**Figure 4:**
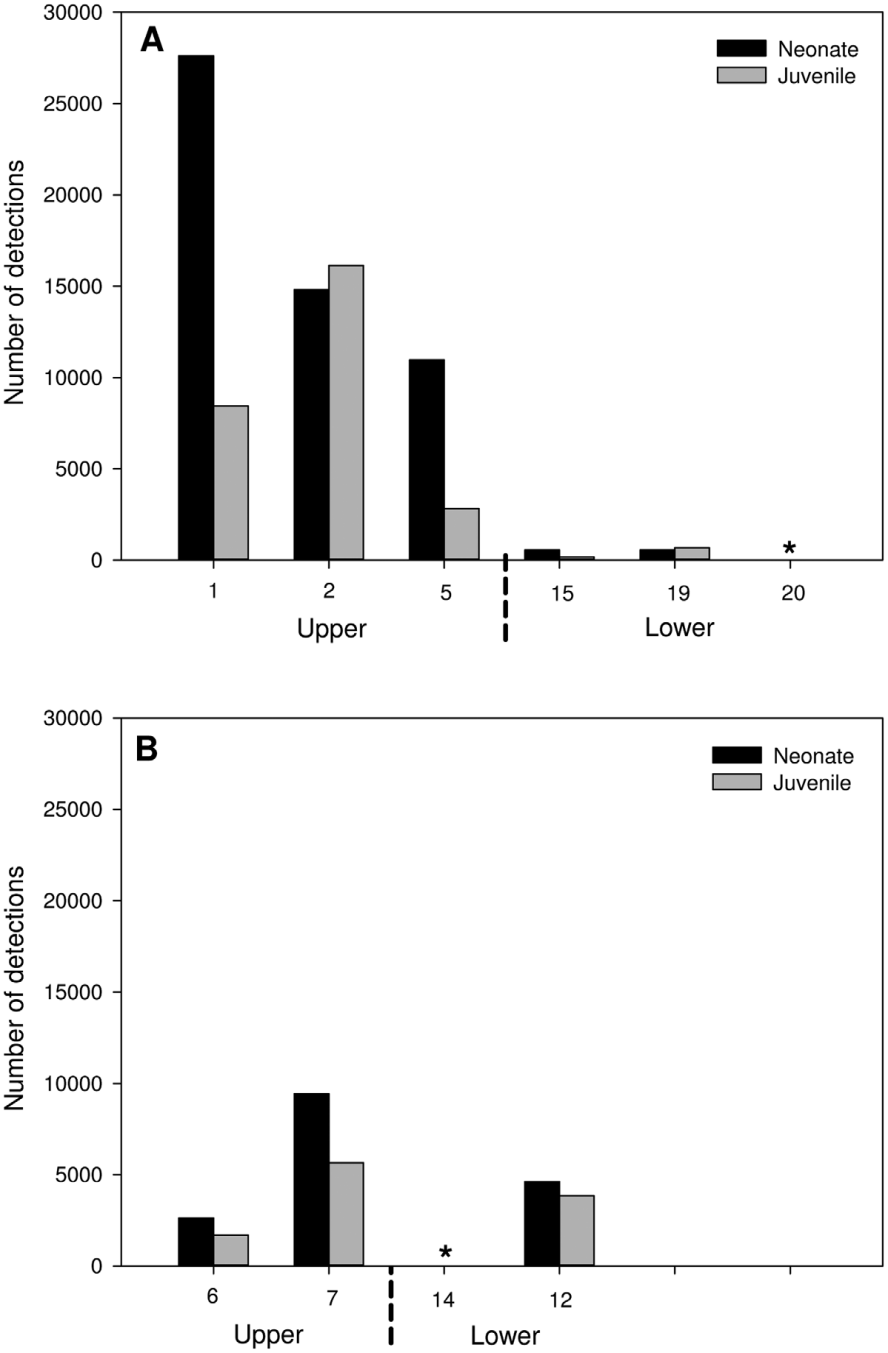
Total detections at individual acoustic receivers for all neonate and juvenile bull sharks tagged with acoustic tags in the Gold Coast system. Numbers on the × axis refer to individual stations (see Figure 1) for river (A) and canal (B) habitats. River numbers correspond to upper river on the left to lower river on the right. Upper canal (6 and 7) and lower canal (14 and 12) are shown accordingly. Asterisk denotes stations with<15 detections.

**Figure 5:**
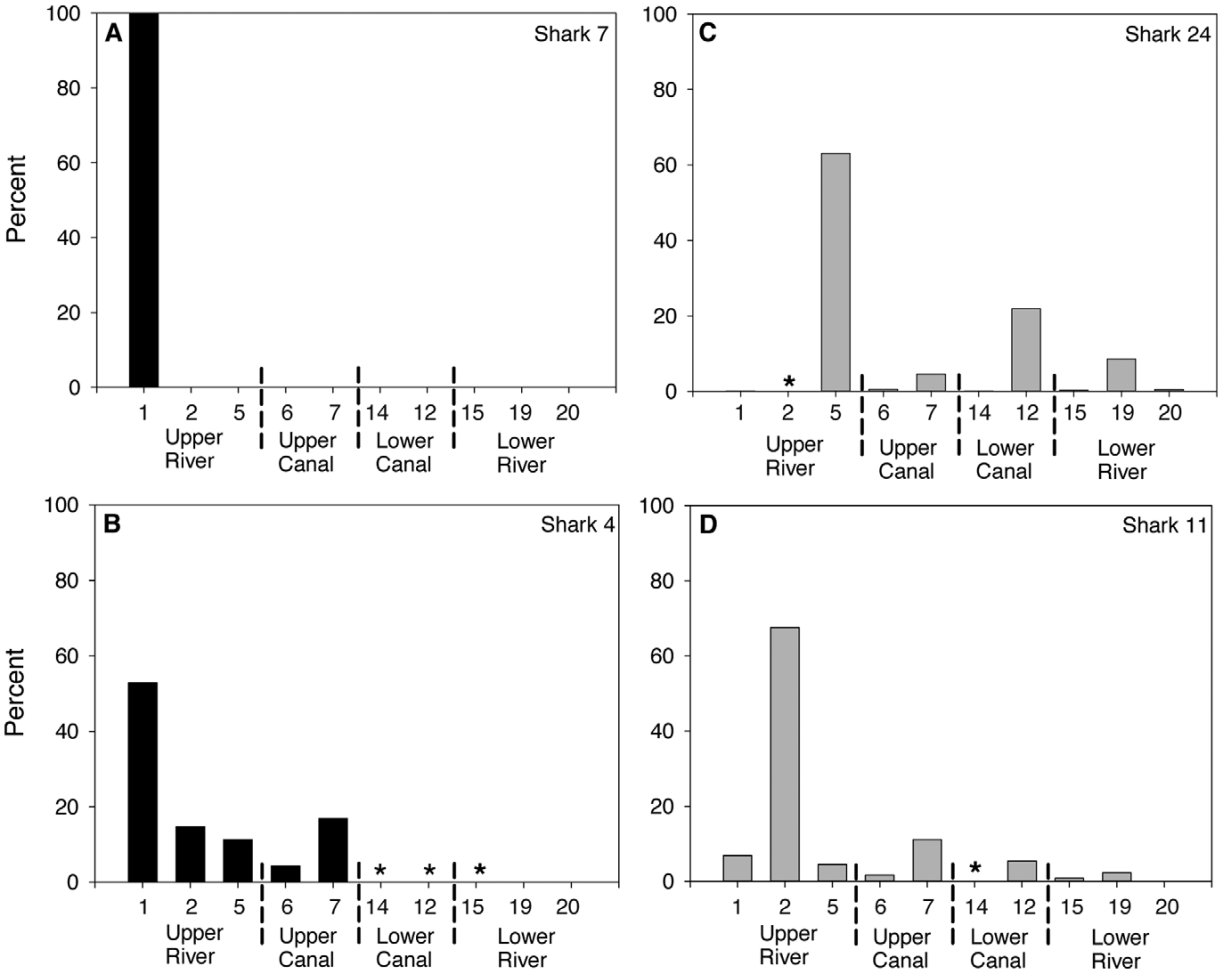
Examples of individual detections across all acoustic receivers for two neonate and two juvenile bull sharks tagged with acoustic tags in the Gold Coast system. Numbers on the × axis refer to individual stations (see Figure 1). Asterisk denotes stations with<5 detections.

**Figure 6:**
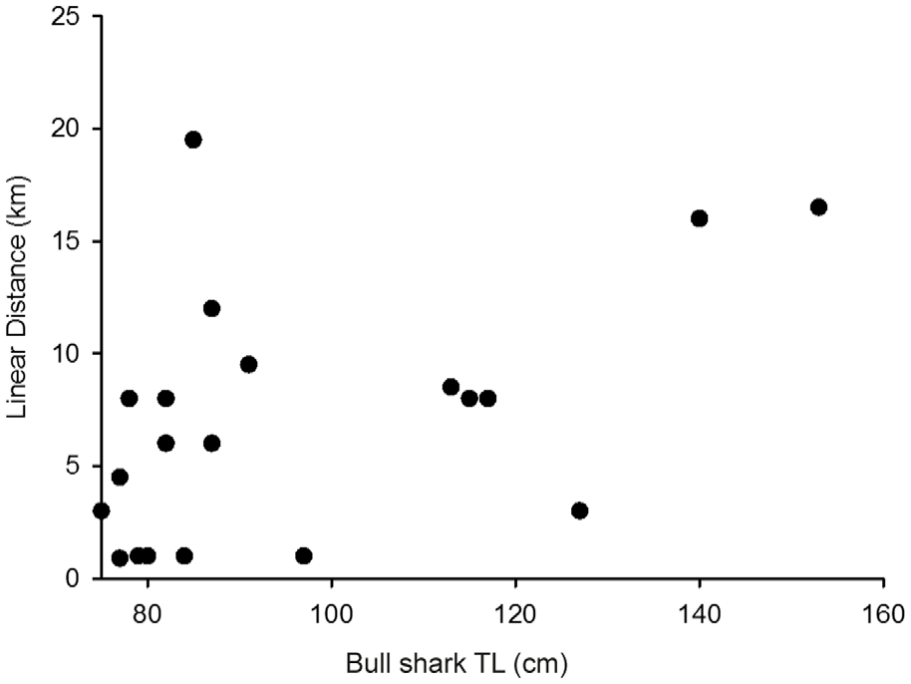
Maximum displacement (km) between detections for bull sharks of varying total lengths (TL) detected on receivers.

Four bull sharks tagged with acoustic tags (1 neonate: male and 3 juveniles: 1 male, 2 female) were caught by recreational fishers. Three were caught in 2007 and one in 2008 (Table 2) and thus the analysis was confined to the detections of the remaining 20 individuals. Despite this, the movements of the nine neonates (7 sharks: 4 male, 3 female tagged in 2007 and 2 sharks: 1 male, 1 female tagged in 2008) and eleven juveniles (6 sharks: 4 male, 2 female tagged in 2007 and 5 sharks: 1 male, 4 female tagged in 2008) produced 110,697 detections on the receiver array with 71% and 29% occurring across 10 of the acoustic receiver sites in the Nerang River and canals, respectively (Fig. 4). Neonates had an average of 8901 detections per individual whereas juveniles had a mean of 3945 detections per individual for the period of acoustic receiver array deployment and were detected during the day and at night. Four neonates tagged in the Nerang River remained in the upper and lower reaches of the river exhibiting pronounced site fidelity at Site 1 as exemplified by Shark 7 (Fig. 5a) with only one individual moving to Site 15 in the lower Nerang River (Table 2). The remaining five neonates, tagged in the Nerang River, stayed mainly within the upper and lower reaches of the river, but also spent brief periods (<20% of their time) in the canals, especially those adjoining the upper reaches of the river (Table 2). The most extensive pattern of movement exhibited by a neonate bull shark was that of Shark 4 (Fig. 5b). This individual exhibited pronounced site fidelity across Sites 1, 2 and 5, spending a combined 79% of its time at these sites. Greater than 20% of its time was spent at Sites 6 and 7 in the upper canals adjoining the Nerang River. This shark also exhibited a rapid excursion (<0.5% of its time) across 8 sites spanning all four habitats before returning to Site 7 in the upper canals (Fig. 5b; Table 2). In contrast, only two of the six juveniles tagged in the Nerang River remained, with the rest spending varying amounts of time in the canals adjoining the upper and lower reaches of the river and in the river *per se* (Fig. 4; Table 2). For example, Shark 24 exhibited pronounced site fidelity (63% of its time) at Site 5 in the upper Nerang River, but also moved to Sites 19 and 12 in the lower river and canals, respectively where it spent 30.5% of its time (Fig. 5c; Table 2). The five juveniles tagged in the canals also spent varying periods of time in the upper and lower reaches of the Nerang River and the adjoining canals (Table 2). These movement patterns were exemplified by Shark 11 (Fig. 5d; Table 2) which exhibited pronounced site fidelity (67.5% of its time) at Site 2 in the upper Nerang River, but also moved between the river and the canals in the upper and lower reaches where it spent its remaining time. When combined, the maximal distances moved by neonate and juvenile bull sharks (Fig. 6) in the Nerang River and the adjoining canals were positively correlated with TL (Pearson’s correlation coefficient: r = 0.73, df = 18, p<0.01).

### Habitat Usage

**Figure 7:**
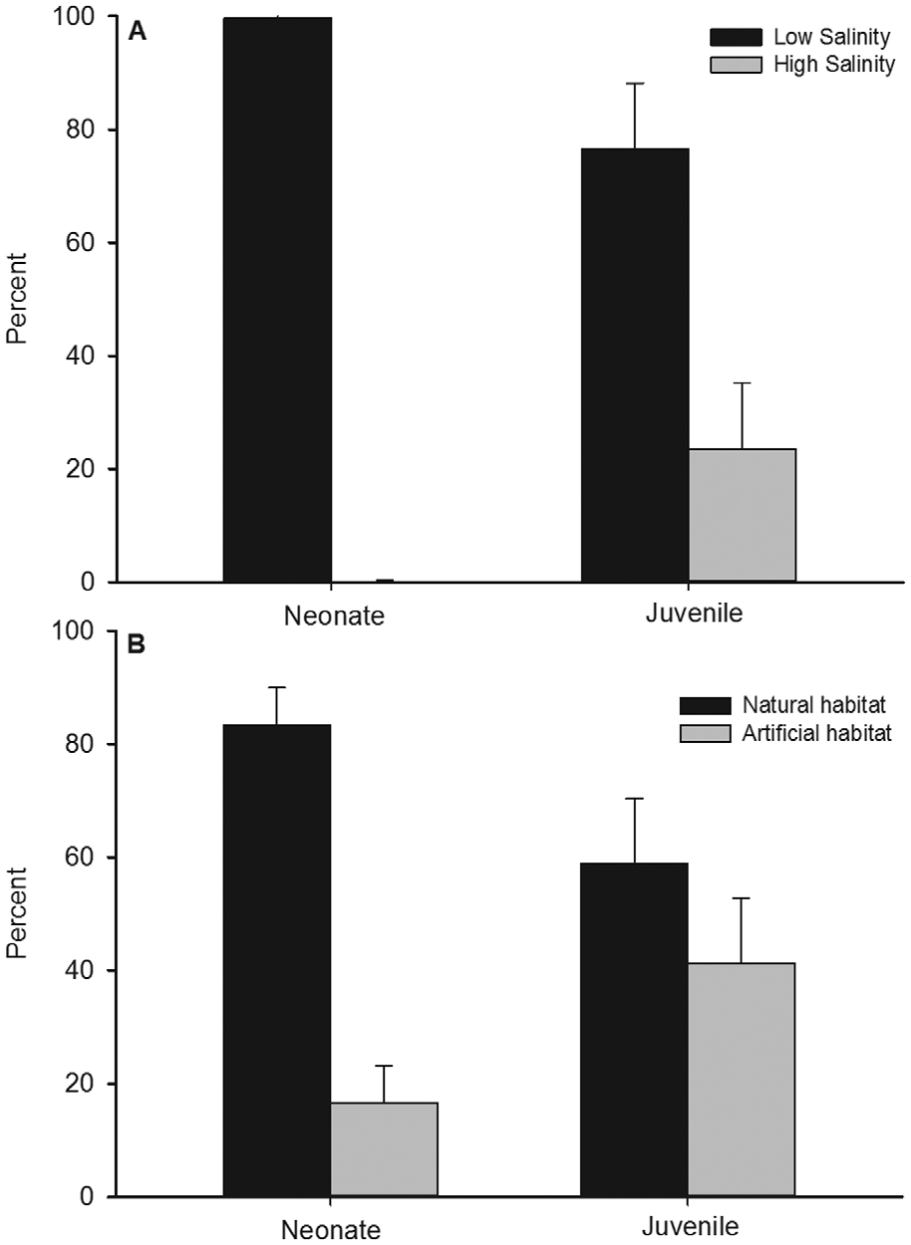
Proportions of time (± SE) bull sharks were detected in differing salinity (A) and habitats (B).

The tagged bull sharks (9 neonates, 11 juveniles) spent the vast majority (>60%; Fig. 7) of their time in the Nerang River and adjoining canals where salinity ranged 6–18 (Fig. 7a;t-test: t = 19.74, df = 18, p<0.001 and t = 2.08, df = 20, p = 0.050). The juveniles moved further afield to areas of higher salinity (19–32) and thus spent significantly less time in areas of lower salinity than did the neonates (Fig. 7a;t-test: t = 2.23, df = 19, p = 0.038). The proportion of time that tagged neonate and juvenile bull sharks occupied the Nerang River and the adjoining canals differed. Neonates spent significantly more time in the Nerang River than in the adjoining canals (Fig. 7b;t-test: t = 3.87, df = 16, p = 0.0014), whereas juveniles spent similar proportions of time in both habitats and these periods did not differ significantly (Fig. 4b;t-test: t = 0.75, df = 20, p = 0.46).

**Figure 8:**
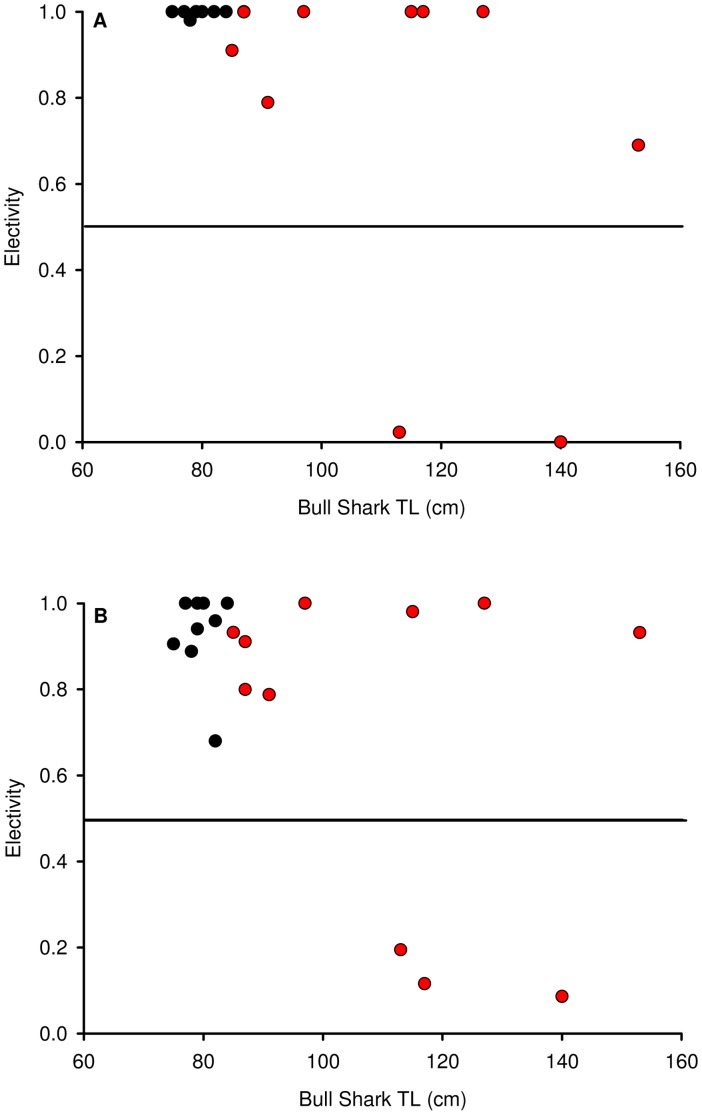
Electivity analyses of bull sharks in the Gold Coast System. Neonate bull sharks are shown by black dots and juveniles with red dots for low salinity electivity (A) and natural habitat (Nerang River) electivity (B).

The electivity analyses for salinity showed that all 9 neonates and 9 of 11 juveniles of varying TL exhibited a pronounced preference for low saline waters (Table 3 and Fig. 8a). The remaining 2 juveniles exhibited a pronounced avoidance of the low saline waters (Table 3 and Fig. 8a). The electivity analyses for natural habitat showed that all 9 neonates and 8 of 11 juveniles of varying TL exhibited a pronounced preference for the Nerang River (Table 4 and Fig. 8b). The remaining 3 juveniles exhibited a pronounced avoidance of the natural habitat (Table 4 and Fig. 8b).

**Table 3:** Salinity electivity analyses for bull sharks in the Gold Coast System.

Salinity (%)	Ontogenetic stage	N	TL range (cm)	Chesson’s α (for selection)	
				Mean (±SE)	Range
6–18	Neonate	9	75–84	0.999 (0.001)	0.999–1.000
	Juvenile	9	85–153	0.976 (0.014)	0.885–0.926
19–32	Neonate	0	–	–	–
	Juvenile	2	113–140	0.963 (0.037)	0.9260–1.000

Selection of waters with particular salinity ranges by neonate and juvenile bull sharks.

**Table 4:** Habitat electivity analyses for bull sharks in the Gold Coast System.

Habitat	Ontogenetic stage	N	TL range (cm)	Chesson’s α (for selection)	
				Mean (±SE)	Range
Natural (River)	Neonate	9	75–84	0.937 (0.032)	0.680–1.000
	Juvenile	8	85–153	0.918 (0.030)	0.788–1.000
Artificial (Canal)	Neonate	0	–	–	–
	Juvenile	3	113–140	0.868 (0.032)	0.806–0.914

Selection of waters with natural or artificial habitats by neonate and juvenile bull sharks.

## Discussion

Bull sharks exhibited differing patterns of distribution, abundance, size-structure, movement, habitat-usage and electivity between the natural (Nerang River) and artificial (canal) habitats. The presence of neonates and juveniles of bull sharks in natural habitat in the upper, lower salinity reaches and larger juveniles in the lower reaches with more variable salinity, was similar to patterns evident in rivers and/or estuaries in Florida, USA [20], [82], [83], [44], Nicaragua [40], Fiji [84] and northern and southeast Queensland, Australia [85], [54], [12]. These patterns can arise via different mechanisms and two competing, explanatory models accounting for the occurrence of neonates in the upper reaches of rivers were summarised by Werry *et al*. [12]. Briefly, Model 1, attributable to Jensen [39] and based on the capture of pregnant females (and no neonates), suggests that parturition of bull sharks occurs in the near-shore areas adjacent to river mouths. Consequently, neonates must swim from the near-shore environment into the upper reaches of rivers. Model 2, proposed by Werry *et al*. [12], suggest that pregnant bull sharks migrate from the nearshore marine environment and into the upper reaches of rivers to give birth. Model 1 predicts that following parturition, neonates would be present in the lower, middle and eventually in the upper reaches as they swim upstream. In contrast, Model 2 predicts that neonates would only be present in the catches from the upper reaches of the river and absent from those in the lower reaches. These contrasting predictions were tested experimentally via the longline surveys (over 3 years) and showed that neonates were absent from the lower and middle reaches and only caught in the upper, low salinity reaches of the Nerang River. The longline surveys also caught a putatively pregnant female in the middle reach of the Nerang River in November 2008 whilst the shark was swimming upstream just prior to the austral spring/summer parturition period. These results provided further support for the explanatory model of Werry *et al*. [12] and further highlight the importance of protecting the upper reaches of tidal rivers as pupping and nursery sites for this species.

### Movements

Spatial distribution patterns integrate biological and environmental influences that ultimately determine habitat-use patterns and movement for sharks in estuarine and coastal environments [86]. Previous studies suggest that environmental factors, particularly salinity for small bull sharks [54], [10], [87], influence metabolism and in turn movements [20]. Small bull sharks display consistent use of estuarine habitats, despite variable environmental conditions, as a strategy to increase survivorship through reduced predation and competition and these areas are often highly productive habitats that provide food [88], [89], [90]. All neonates in our study were caught and tagged in the upper reaches of the Nerang River where recruitment occurred, as has been documented in numerous previous studies [20], [54], [91]. We found that tagged neonate bull sharks displayed consistent use of the upper reaches of the Nerang River with limited overall movements and only small occasional movements into the adjoining upper canals before returning to the upper river. The proportion of time spent in the natural causeway of the Nerang River compared to the artificial habitat provided by the canals differed overwhelmingly. While bull sharks adapt well to physiological disturbances [89], extensive development adjacent to shark nursery areas has reduced the survival of neonatal lemon sharks [92] and decreased habitat quality could affect bull sharks in unforeseen ways. In the GCS, the differences in use of natural and artificial habitats by bull sharks was likely influenced by the spatial and temporal variation in the physical and hydrological characteristics of the river and the adjoining canals [23], [60], [57]. While the species composition of the estuarine fish community in the canals mirrors that found in the river [61], there are often lower abundances of prey and small fish mainly associate with artificial structures, e.g. jetty pilings for refuge rather than food [93] and this may affect predatory search images [94] and reduce movements into the canals. The mangrove-fringed upper reaches of the river, likely provide favourable habitats for neonates and hence their movements were restricted to this habitat [23], [60]. With ontogenetic increases in size, the initial size-limited refuges would be outgrown, necessitating movements to other habitats with appropriate refuges. To this end, the canals could provide a greater number and range of refuges from large predators (e.g. adult conspecifics [95], [12]) owing to their spatial extent and physical complexity and this, in turn, could enhance the movements of larger juvenile bull sharks into the canals.

The long-term use of the upper reaches of the Shark River Estuary (Everglades, Florida, USA) by small bull sharks was hypothesised as a predation avoidance behaviour rather than one triggered by access to food resources [83]. However, the presence of sharks at smaller spatial and temporal scales was considered to be driven by abiotic conditions [83]. Pillans and Franklin [54] found an increase in the size of bull sharks with increasing salinity in the Brisbane River, Australia and Heupel and Simpfendorfer [10] suggested young-of-the year and juvenile bull sharks move to remain with optimal salinity and temperature based on findings in the Caloosahatchee River of southwest Florida, USA. While the pattern of small juvenile and neonate bull sharks occurring in the top reaches of river systems and concentrated at one location appears to be consistent across different studies, the likely drivers of size-based segregation of bull sharks may differ between river systems.

The movement of bull sharks at different ontogenetic stages throughout natural and artificial habitats in the GCS could be due to greater energetic requirements with size or age. Neonates and juvenile bull sharks use estuaries as nursery grounds where mullet (*Mugil* spp.), a major component of the diet of bull sharks in this size range [22], are in high abundance. Bull sharks also exhibit ontogenetic changes in diet and prey size [41], which commonly accompany changes in foraging tactics and habitat, probably as a strategy to increase net rate of energy gain with increase in size or age.

Juveniles of various shark species commonly exhibit strong site fidelity (e.g. [96]), whereas larger (i.e. older) sharks tend to range over much wider areas [97], [44]. With increasing body size, movements by bull sharks between habitats along the freshwater-estuarine-marine continuum also increase [12]. This pattern was evident for bull sharks in the current study with the neonates mainly occupying mangrove-lined areas with low salinity in the upper reaches of the Nerang River and only entering adjoining canals with similar low salinities for brief periods. In contrast, the larger juveniles moved throughout the Nerang River and adjoining canals spending similar amounts of time in both habitats across a range of salinities. Several studies suggest that organisms select their habitat on the basis of food availability (e.g. [98], [99]), but physical structure and hydrological characteristics of the canals may also influence the movement of large juveniles, sub-adults and adults into and out of these systems.

### Habitat Electivity

A clear preference for low salinity (6–18) waters and the Nerang River over the adjoining artificial habitat was evident for neonates and almost all juveniles. Preference for low salinity waters is well established for neonate bull sharks [10]. However, the avoidance of the adjoining canals in the upper reaches of the Nerang River by neonates suggests preference for natural habitat in spite of the additional habitat provided by the canals. Avoidance of the Nerang River occurred in several larger juveniles (113–153 cm TL) and these individuals also avoided low salinity waters. Reduced physiological constraints and susceptibility to predation likely enable larger bull sharks to move over a wider range of habitats and thus the canals adjoining the lower reaches of rivers are more likely to support larger bull sharks. In estuaries, bull sharks are potentially more vulnerable to habitat modifications compared to their oceanic counterparts [100], [37], [56]. Moreover, riverine and estuarine habitats are spatially constrained, have limited volume and their physico-chemical properties can vary widely because of multiple inputs into the system [56]. Our data clearly demonstrate that canals are not the preferred habitat of neonate bull sharks.

### Natural vs Artificial Habitat Comparisons

Humans frequently exert rapid, large-scale effects on their surrounding environment in coastal areas and these include the modification of waterways, declines and/or loss of riparian vegetation (e.g. mangroves) and the construction of canal systems that provide artificial habitat [101], [15], [16]. Canals and the associated residential infrastructure facilitate increased human-use and increase the likelihood of anthropocentric impacts on adjoining coastal waterways. Diffuse and point-source impacts can occur via: fishing, anti-fouling paints from boats, increased erosion from the wake of vessels, urban run-off and its associated pollutants. With the ever-increasing urbanisation of the coastal zone [102], various interactions with the natural environment including dangerous sharks such as the bull shark, are likely to increase. Given this human demographic shift and the construction of residential canal estates on a global scale [16], it is surprising that the use of natural and artificial habitats by different ontogenetic stages of bull sharks has not previously been investigated and compared in detail. Many studies have confirmed the importance of coastal areas to bull sharks (e.g. [84], [87]) and the significance of natural riverine habitat for neonate bull shark populations ([52], [12]). With studies suggesting declines in bull shark populations worldwide [103], [42], the need to maintain and/or protect habitat important to neonates from anthropogenic impacts will increase in the future.

While much of the previous ecological work on bull sharks has concentrated on natural systems, artificial habitats associated with the lower reaches of urbanised rivers or estuaries may provide increasingly important areas for large juvenile bull sharks. In the GCS the spatial extent of natural and artificial habitat in the upper reaches was similar. In contrast spatial extent of the combined natural and artificial habitat was about 3.5 times greater in the lower reaches with the artificial habitat (canals) providing almost 60% more area than natural habitat. In spite of this, there was a pronounced preference by bull sharks for natural over artificial habitats. We hypothesise that this pattern is due to the presence of riparian vegetation (predominantly the grey mangrove, *Avicennia marina*, in the GCS), and well-established tidal flows which greatly contrast with the canals where riparian vegetation is almost non-existent and tidal flow is greatly modified resulting in the deoxygenation of the bottom layers, a strong deterrent to demersal species such as the bull shark [83].

Like many studies, this research was limited by severe logistic constraints due to the engineering complexity and spatial extent of the GCS [58], [60]. This meant that the sampling effort required to obtain adequate and representative data for testing our hypotheses prevented replication of the natural/artificial habitat contrast in another, similar system in QLD. Consequently, determining whether the patterns observed in the GCS are evident in similar systems in Australia and elsewhere in the world needs to be investigated in the future. Given the inherent complexity of these co-joined natural/artificial systems, cost-effective and efficacious assessments of the impacts on bull sharks will necessitate that further studies consider multiple-agency collaborations, adopt a multi-disciplinary focus, and ensure consistent sampling approaches at different spatial and temporal scales to avoid pseudoreplication [104] and have sufficient statistical power to detect ecologically important changes [105], [106], [107].

### Global Implications for Conservation and Management

The decline in populations of large top-level predators is of growing global concern and mainly attributable to legal and illegal fishing [42], [5], and shark-finning [6]. We argue however, that the neonatal and juvenile stages of bull sharks, which together constitute the first 5 to 6 years of the shark’s life-history [46], [12], are probably very vulnerable to habitat modification associated with the urbanisation of the coastal fringe (*sensu* Yapp [102]). As such, it is likely that this surreptitious impact has contributed to the population decline of bull sharks and maximising the survival of neonates and juveniles will be critical to the future replenishment of the populations globally. Moreover, we suggest that future research should focus on pregnant females close to parturition, the resulting neonates and juveniles, their associated habitats and subsequent ontogenetic changes in habitat-use [12]. This would provide a more cost-effective and efficacious approach to long-term conservation given that the habitats utilised by neonate and juveniles are relatively restricted compared to adult bull sharks that occupy open, offshore waters [108].

Continued urbanisation will likely affect bull shark populations in differing ways. First, as bull sharks are potentially philopatric with pregnant females returning to pup in the same river used during their own neonatal phase [109], it will be important to determine whether the natural features of these systems become degraded with: (1) urbanisation over time, and/or (2) following the construction of adjoining residential canal estates. With further degradation, the value of the natural habitat as a nursery area may be reduced leading to the abandonment of the site and a demographic shift in the population to a more pristine, natural system elsewhere. Second, as preferred natural habitats become less available bull sharks may occupy artificial habitats, including canal systems, for greater periods of time especially if their “naturalness” is enhanced by the development of stands of mangroves (via deliberate planting or natural seed-set) at sites with appropriate physico-chemical conditions (e.g. reduced salinity for neonates). Increased use of canals by bull sharks may have advantages and disadvantages for the shark and human users of these systems. For example, recreational fishers target bull sharks in the GCS [73] and fishing-related mortality could further exacerbate the effects of habitat loss. Alternatively, the greater use of canals by bull sharks could lead to more frequent attacks on swimmers compared to those in the past [53]. Future work will need to test predictions emanating from these possible outcomes and then be used to inform conservation initiatives and the management of land-use and shark-human interactions.

In conclusion, our study has shown that the natural Nerang River was preferred over artificial (canal) habitat by neonate bull sharks. These results are particularly relevant globally as the species is on a collision-course with coastal development as exemplified by the anastomosing of natural and artificial habitats. Populations of bull sharks are also not immune from the tyranny of small decisions and their cumulative effects that lead to the further degradation of natural habitats [110]. The inevitable conflicts between ecocentric and anthropocentric viewpoints will necessitate compromises in land-use and seascape planning. Hopefully, these recognise the ecological role and benefits of maintaining top-order predators, such as the bull shark, in the riverine/estuarine regions of the coastal fringe and more widely in the adjoining continental shelf waters.
